# Threshold effect and age interaction of TyG index on diabetes incidence in normolipidemic population: a multicenter cohort study

**DOI:** 10.3389/fendo.2025.1645344

**Published:** 2025-11-03

**Authors:** Hongchao Chen, Qi Chen, Li Xia, Songyao Lu, Xiaohong Cai, Xudong Huang, Juan Wu, Weihan Lin

**Affiliations:** ^1^ Department of Clinical Laboratory, Jieyang People’s Hospital, Jieyang, Guangdong, China; ^2^ Department of Hematology, The Third Affiliated Hospital of Sun Yat-sen University & Sun Yat-sen Institute of Hematology, Guangzhou, Guangdong, China; ^3^ Department of Respiratory and Critical Care Medicine, Jieyang People’s Hospital, Jieyang, Guangdong, China

**Keywords:** triglyceride-glucose index, diabetes, age interaction, cohort study, nonlinear relationship

## Abstract

**Background:**

Although the triglyceride-glucose (TyG) index-diabetes association has been widely studied, its relationship in normolipidemic populations remains poorly understood.

**Methods:**

A retrospective cohort of 60,103 normolipidemic Chinese adults was included from routine health screening programs conducted across 32 healthcare institutions in China. Data collection included demographic characteristics, anthropometric measurements, serum biochemical parameters, smoking and alcohol consumption history, and family history of diabetes. We employed multivariable Cox regression, restricted cubic spline analysis, threshold effect analysis, stratified analysis, and interaction tests to comprehensively assess the association between the TyG index and incident diabetes.

**Results:**

Multivariable-adjusted Cox regression revealed a robust positive association between the TyG index and incident diabetes in normolipidemic subjects (HR: 10.10, 95% CI: 7.94–12.84, *P* < 0.001). Restricted cubic spline analysis detected a nonlinear relationship, with a critical threshold at TyG ≥ 8.53, beyond which diabetes risk increased exponentially (HR: 51.84, 95% CI: 24.83–108.24, *P* < 0.001). Despite consistent findings across subgroups, a significant interaction with age was detected (*P* for interaction < 0.05).

**Conclusions:**

In normolipidemic individuals, the TyG index demonstrated a nonlinear positive association with diabetes risk, particularly above 8.53. It can serve as an early warning signal for diabetes risk in normolipidemic individuals, facilitating personalized prevention strategies for diabetes prevention and control.

## Introduction

Diabetes mellitus is a highly prevalent chronic metabolic disorder worldwide. It is projected that by 2040, the global diabetic population will reach 642 million, with 60% of cases occurring in Asia (1, 2). The situation is particularly severe in Southeast Asia, where the mortality rate among its 82 million diabetic patients is as high as 14% (3). Epidemiological data reveal a dual high-prevalence trend in both developed countries and developing countries. China, the country with the largest diabetic population globally, reported an adult prevalence rate of 11.2% in 2020 (4). Beyond dysregulated glucose metabolism, diabetes leads to multisystem complications, including cardiovascular diseases, diabetic nephropathy, and retinopathy, which significantly impair patients’ quality of life, reduce life expectancy, and impose a substantial burden on healthcare systems. In 2021, China ranked second globally in diabetes-related healthcare expenditures, underscoring the urgency of disease prevention and control (5). Notably, diabetes is highly preventable. Studies have demonstrated that lifestyle modifications, such as balanced nutrition, regular physical activity, and early screening interventions, can effectively reduce diabetes risk and slow disease progression (6).

The development of diabetes mellitus results from a complex interplay of multiple pathological factors, among which impaired insulin sensitivity and defective pancreatic β-cell function serve as the central pathogenic mechanisms (7). Additional contributing factors include genetic predisposition, chronic inflammation, oxidative stress, and gut microbiota dysbiosis (8). Notably, insulin resistance typically precedes β-cell dysfunction and persists throughout the disease course, currently recognized as the primary and most critical initiating factor in diabetes pathogenesis. Dyslipidemia represents a well-established risk factor, with hypertriglyceridemia and reduced high-density lipoprotein cholesterol (HDL-C) levels serving as characteristic markers of insulin resistance (9). Mechanistically, elevated triglycerides exacerbate insulin resistance by activating inflammatory signaling pathways and inhibiting insulin receptor substrate phosphorylation (10). While the detrimental effects of dyslipidemia on metabolic and cardiovascular diseases are well-documented, the diabetes risk factors in normolipidemic individuals remain less clearly defined. Even within normal lipid ranges, these individuals may harbor other underlying metabolic disturbances - including insulin resistance, chronic low-grade inflammation, and oxidative stress - that potentially elevate diabetes risk. This highlights the importance of evaluating diabetes susceptibility in normolipidemic populations, particularly regarding insulin resistance assessment. The hyperinsulinemic-euglycemic clamp remains the gold standard for insulin resistance measurement. However, its widespread clinical application is limited by substantial costs, time-consuming procedures, and requirements for specialized equipment and technical expertise, currently restricting its use primarily to research settings (11).

The triglyceride-glucose (TyG) index, calculated from fasting plasma glucose (FPG) and triglyceride (TG) levels, has emerged as a simple, cost-effective, and reliable surrogate marker for insulin resistance (12). Growing evidence has highlighted its research value in metabolic and cardiovascular diseases in recent years (11, 13–16). Notably, multiple studies have demonstrated that the TyG index outperforms established diabetes risk predictors, including the homeostasis model assessment of insulin resistance, oral glucose tolerance test, and triglyceride to high-density lipoprotein cholesterol ratio, in predicting type 2 diabetes mellitus (17–19). Substantial evidence also indicates a strong association between the TyG index and diabetes risk, suggesting its potential as an early warning indicator for diabetes development (20, 21). However, most existing studies have focused on general populations or high-risk individuals with dyslipidemia (22, 23). Whether the TyG index maintains its predictive value for diabetes risk in normolipidemic populations remains unclear and warrants further investigation.

Our study aims to examine the relationship between the TyG index and diabetes incidence in individuals with normal lipid profiles, thereby evaluating its clinical utility in this specific population. The results could support the development of more effective screening and prevention protocols for diabetes in individuals with apparently normal lipid profiles.

## Method

### Data source

The data for our study were obtained from the Dryad Digital Repository (https://datadryad.org), a specialized platform for medical and health science research data. Dryad database maintains strict adherence to ethical guidelines and privacy protection principles throughout its data storage and sharing processes. The dataset utilized in our study has been fully anonymized, with all personally identifiable information removed to ensure participant confidentiality. As such, this secondary analysis of de-identified data did not require additional informed consent from participants, in accordance with standard research ethics protocols for publicly available, non-identifiable datasets. The open-access nature of these data supports their legitimate use by researchers for secondary analytical purposes.

### Study population

**Figure 1:**
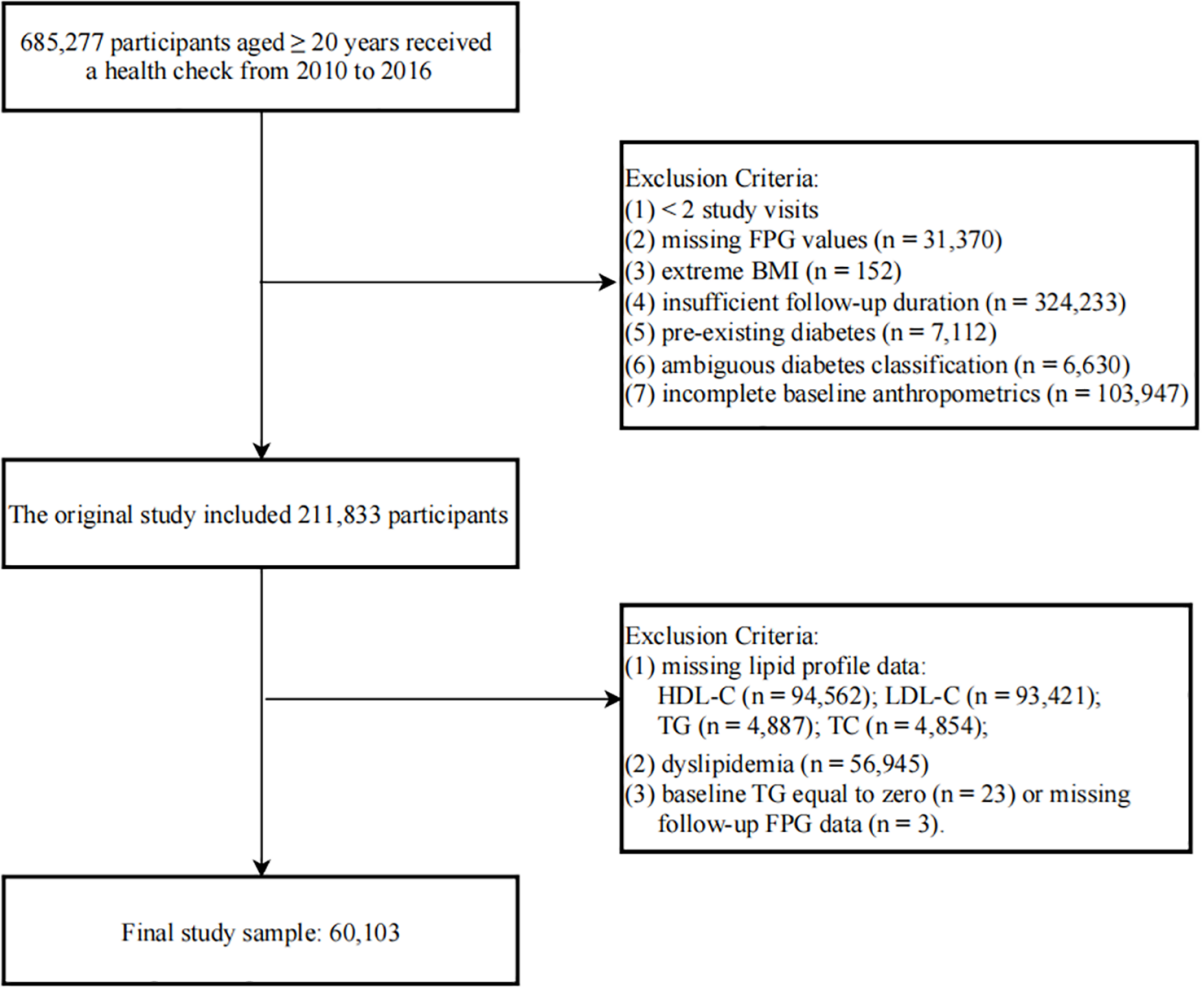
Flowchart outlining the structure of the study.

In our study, we analyzed medical examination data provided by the Rich Healthcare Group, which included adults aged 20 years and older who underwent a minimum of two health check-ups between 2010 and 2016 at 32 sites across 11 cities in China. The initial exclusion criteria, as outlined by Chen et al. (24), were as follows (1): absence of data on body weight, height, or sex (n = 103,947) (2); absence of FPG data (n = 31,370) (3); extreme body mass index (BMI) values, defined as < 15 kg/m² or > 55 kg/m² (n = 152) (4); individuals with an inter-visit interval of less than 2 years (n = 324,233) (5); a baseline diagnosis of diabetes (n = 7,112) (6); undetermined diabetes status (n = 6,630). Following these exclusions, the initial cohort consisted of 211,833 participants. From this cohort, we further excluded individuals with (1): missing lipid profile data, specifically HDL-C (n = 94,562), low-density lipoprotein cholesterol (LDL-C) (n = 93,421), TG (n = 4,887), and total cholesterol (TC) (n = 4,854) (2); dyslipidemia (n = 56,945); and (3) baseline TG equal to zero (n = 23) or missing follow-up FPG data (n = 3). After implementing all exclusion criteria, our final analytical sample comprised 60,103 participants (**Figure 1**).

### Missing value handling

The dataset exhibited variable-specific missing data patterns, with missingness proportions as follows: smoking status and alcohol consumption shared identical missing rates (73.47%, n = 44,157 each), followed by aspartate aminotransferase (AST; 56.85%, n = 34,169). Other variables showed minimal missingness: alanine aminotransferase (ALT; 0.40%, n = 242), blood urea nitrogen (BUN; 2.23%, n = 1,343), serum creatinine (Scr; 1.35%, n = 814), and blood pressure measures (systolic and diastolic both 0.01%, n = 6 each). The pattern and proportion of missing data for all variables are presented in **Supplementary Table 1**.

For continuous variables with more than 50% missing data, such as AST, we employed tertile categorization, designating missing values as “NA”. Similarly, categorical variables with over 50% missingness, including smoking and alcohol status, were assigned “NA”. Continuous variables with less than 5% missingness were subjected to multiple imputation using chained equations (25). This stratified methodology was designed to optimize analytical validity while maintaining the integrity of the dataset, despite the presence of substantial incomplete observations. The primary outcome analyses in our study were conducted using the imputed dataset.

### Data collection

The study protocol involved systematic acquisition of demographic characteristics (age, sex), lifestyle parameters (tobacco use, alcohol consumption patterns), and familial diabetes history through standardized questionnaires. Certified personnel conducted anthropometric assessments encompassing height, body mass, and blood pressure measurements. During each clinical assessment, fasting venous blood samples (minimum 10-hour fasting duration) were obtained for comprehensive biochemical profiling, including quantitative analysis of lipid profile (HDL-C, LDL-C, TC, TG), FPG, renal function markers (BUN, Scr), liver enzymes (ALT, AST). The observation period spanned from baseline evaluation to either study termination or incident diabetes diagnosis.

### Definition

The TyG is a marker based on FPG and TG levels, used to assess insulin resistance. Its calculation formula is: TyG = ln [FBG (mg/dL) × TG (mg/dL)]

Diabetes was defined as either FPG ≥ 7.0 mmol/L or self-reported diabetes (26).

Prediabetes was defined as FPG between 5.6 and 6.9 mmol/L and no history of diabetes (26).

Hyperlipidemia is defined as an abnormal elevation of blood lipids, including:

TC > 5.2 mmol/L, TG > 1.7 mmol/L, LDL-C > 3.12 mmol/L or HDL-C < 1.03 mmol/L (27).

Normal blood pressure is defined as systolic blood pressure (SBP) < 120 mmHg and diastolic blood pressure (DBP) < 80 mmHg (28).

### Statistical analysis

Continuous variables were expressed as mean ± standard deviation or median (interquartile range) based on distributional characteristics, while categorical variables were presented as frequencies (percentages). Inter-group comparisons across TyG index quartiles were performed using ANOVA or Kruskal-Wallis tests for continuous measures and chi-square tests for categorical variables. The standardized mean difference (SMD) was calculated to quantify covariate balance between groups, with an absolute SMD >0.1 indicating potential imbalance.

Survival analyses were conducted using Kaplan-Meier estimators stratified by the TyG quartiles with log-rank tests. Multivariable Cox proportional hazards models were constructed to evaluate independent associations. Confounders were selected based on clinical judgment and previous scientific literature (29, 30). We constructed a total of 4 models for the analysis: No covariates were adjusted for Model 1. Model 2 adjusted for age and sex. Model 3 further accounted for BMI, SBP, DBP, ALT, AST, BUN, and Scr base on model 2. Model 4 designated as the main model, included all previous adjustments and additionally accounted for family history of diabetes, smoking status, and alcohol consumption. Effect estimates were expressed as hazard ratios (HR) with 95% confidence intervals (95% CI).

We categorized TyG into quartiles to assess the trend in its association with the diabetes incidence. This approach allowed us to verify the results obtained when considering TyG as a continuous variable and to explore potential non-linear relationships. To evaluate the robustness of our findings, we conducted sensitivity analyses excluding participants with any history of tobacco use or alcohol consumption. Additionally, we validated the stability of imputed results using the original dataset. Finally, we performed separate analyses among individuals with normoglycemia and those with prediabetes at baseline to evaluate the association between the TyG index and the risk of incident diabetes across different glycemic statuses. Moreover, Subgroup analyses were conducted, evaluating TyG-diabetes associations across clinically relevant partitions (age [< 50/≥ 50 years old] (31), sex, BMI [< 24/≥ 24 kg/m²] (32), blood pressure status, and family history of diabetes). Interaction effects were quantified using multiplicative terms, with statistical significance determined via Wald tests.

To further investigate the relationship between the TyG index and diabetes risk, we utilized a restricted cubic spline (RCS) model to generate smoothed curves that visualize the potential non-linear dose-response association. In this model, TyG was treated as a continuous variable, utilizing four knots at the 5th, 35th, 65th, and 95th percentiles. Threshold effects were evaluated through two-piecewise binary logistic regression model, with the statistical significance of inflection points determined by likelihood ratio tests.

For subgroups exhibiting statistically significant interaction effects, comprehensive assessments of effect modification were conducted. We utilized a dual-stratification analytical framework (1): Age stratification with a cutoff at 50 years old (2); Stratification of the TyG index using thresholds determined through RCS analysis. Utilizing this stratification framework, participants were categorized into four mutually exclusive subgroups for interaction analysis (1): Younger age (< 50 years old) with a low TyG index (below the RCS-derived threshold) (2); Younger age (< 50 years old) with a high TyG index (above the RCS-derived threshold) (3); Older age (≥ 50 years old) with a low TyG index (4); Older age (≥ 50 years old) with a high TyG index. We conducted a systematic evaluation of both multiplicative and additive interactions to thoroughly investigate the relationship between age and the TyG index. Multiplicative interaction refers to whether the combined effect of two factors exceeds the product of their individual effects, whereas additive interaction evaluates whether their joint effect surpasses the sum of their separate effects. To assess multiplicative interaction, we incorporated a product term (age group × TyG category) into multivariable logistic regression models. A statistically significant product term (*P* < 0.05) signifies effect modification on the multiplicative scale. Additive interaction was assessed utilizing two well-established metrics: the relative excess risk due to interaction (RERI) and the attributable proportion (AP), with statistical significance evaluated through 95% CI. The RERI measures the incremental disease risk attributable to the synergistic interaction between TyG index and diabetes incidence, beyond the anticipated additive effects of their individual contributions. The AP denotes the proportion of disease incidence among individuals exposed to both risk factors that is specifically attributable to their interaction, expressed as a percentage of the overall risk.

### Statistical software

All statistical analyses were executed using R Statistical Software (Version 4.2.2, http://www.R-project.org, The R Foundation) and the Free Statistics analysis platform (Version 2.1.1, Beijing, China, http://www.clinicalscientists.cn/freestatistics). Free Statistics is a software package that provides intuitive interfaces for common analyses and data visualization, utilizing R as its underlying statistical engine and employing a graphical user interface developed in Python. A two-sided p-value of less than 0.05 was deemed statistically significant.

## Results

### Baseline demographic and clinical profiles

As shown in **Table 1**, the longitudinal cohort (N = 60,103) demonstrated 732 incident diabetes cases during a mean follow-up duration of 3.06 years. Significant gradient patterns across TyG quartiles were observed for all covariates (*P* < 0.001 for all except family history of diabetes). Progressive elevation of age, BMI, blood pressure parameters, and lipid profiles (TG, TC, LDL-C) accompanied ascending TyG quartiles, while HDL-C exhibited an inverse pattern. The sex distribution shifted from a predominance of females (72.6%) in the first TyG quartile to a predominance of males (63.4%) in the fourth quartile. Additionally, hepatic enzyme levels and the proportions of current smokers and individuals consuming alcohol increased proportionally across TyG quartiles. Notably, the prevalence of diabetes rose from 0.2% in the first TyG quartile to 3.2% in the fourth quartile, indicating a 16-fold increase. Most variables demonstrating statistically significant differences in **Table 1** (excluding BUN, smoking or drinking status, and family history of diabetes) exhibited SMD values exceeding 0.1, indicating substantial baseline imbalances across TyG index quartile groups.

**Table 1:** Baseline characteristics of the participants stratified by TyG quartiles.

TyG quartiles	Total	Quartile 1	Quartile 2	Quartile 3	Quartile 4	*P*-value	SMD
		≤ 7.78	> 7.78, ≤ 8.09	> 8.09, ≤ 8.41	> 8.41		
Participants (Numbers)	60,103	15,021	15,024	15,029	15,029		
Age (Years)	41.0 ± 11.9	37.7 ± 9.6	39.7 ± 11.1	41.8 ± 12.3	44.9 ± 13.2	< 0.001	0.342
Sex						< 0.001	0.419
male	27,347 (45.5)	4,109 (27.4)	5,992 (39.9)	7,719 (51.4)	9,527 (63.4)		
female	32,756 (54.5)	10,912 (72.6)	9,032 (60.1)	7,310 (48.6)	5,502 (36.6)		
BMI (kg/m^2^)	22.4 ± 3.1	21.1 ± 2.5	21.9 ± 2.8	22.6 ± 2.9	24.0 ± 3.2	< 0.001	0.538
SBP (mmHg)	116.1 ± 15.6	111.3 ± 13.7	114.3 ± 14.8	117.2 ± 15.6	121.7 ± 16.4	< 0.001	0.376
DBP (mmHg)	72.2 ± 10.4	69.3 ± 9.5	71.1 ± 9.9	72.9 ± 10.2	75.5 ± 10.8	< 0.001	0.333
FPG (mmol/L)	4.9 ± 0.6	4.6 ± 0.5	4.8 ± 0.5	4.9 ± 0.5	5.2 ± 0.5	< 0.001	0.570
TC (mmol/L)	4.3 ± 0.5	4.1 ± 0.5	4.3 ± 0.5	4.3 ± 0.5	4.4 ± 0.5	< 0.001	0.309
TG (mmol/L)	0.9 ± 0.3	0.5 ± 0.1	0.7 ± 0.1	1.0 ± 0.1	1.4 ± 0.2	< 0.001	3.268
HDL-C (mmol/L)	1.4 ± 0.2	1.5 ± 0.3	1.5 ± 0.3	1.4 ± 0.2	1.3 ± 0.2	< 0.001	0.342
LDL-C (mmol/L)	2.4 ± 0.4	2.3 ± 0.4	2.4 ± 0.4	2.4 ± 0.4	2.5 ± 0.4	< 0.001	0.305
BUN (mmol/L)	4.6 ± 1.2	4.5 ± 1.1	4.5 ± 1.2	4.6 ± 1.2	4.7 ± 1.2	< 0.001	0.081
Scr (μmol/L)	68.2 ± 15.5	63.7 ± 13.8	66.8 ± 14.8	69.6 ± 15.4	72.8 ± 16.5	< 0.001	0.332
ALT (U/L)	15.6 (11.8, 22.4)	13.1 (10.5, 18.0)	14.5 (11.0, 20.4)	16.3 (12.0, 23.2)	19.3 (14.0, 28.0)	< 0.001	0.237
AST (U/L)	21.0 (17.9, 25.0)	20.0 (17.0, 23.0)	20.0 (17.2, 24.0)	21.0 (18.0, 25.0)	22.2 (19.0, 26.9)	< 0.001	0.147
Smoking status						< 0.001	0.084
Current smoker	2,366 (3.9)	274 (1.8)	478 (3.2)	678 (4.5)	936 (6.2)		
Ever smoker	564 (0.9)	69 (0.5)	124 (0.8)	158 (1.1)	213 (1.4)		
Never smoker	13,016 (21.7)	3,182 (21.2)	3,309 (22.0)	3,324 (22.1)	3,201 (21.3)		
Not recorded	44,157 (73.5)	11,496 (76.5)	11,113 (74.0)	10,869 (72.3)	10,679 (71.1)		
Drinking status						< 0.001	0.076
Current drinker	318 (0.5)	29 (0.2)	54 (0.4)	90 (0.6)	145 (1.0)		
Ever drinker	2,438 (4.1)	347 (2.3)	535 (3.6)	698 (4.6)	858 (5.7)		
Never drinker	13,190 (21.9)	3,149 (21.0)	3,322 (22.1)	3,372 (22.4)	3,347 (22.3)		
Not recorded	44,157 (73.5)	11,496 (76.5)	11,113 (74.0)	10,869 (72.3)	10,679 (71.1)		
Family history of diabetes						0.741	0.007
No	58,803 (97.8)	14,701 (97.9)	14,713 (97.9)	14,694 (97.8)	14,695 (97.8)		
Yes	1,300 ( 2.2)	320 (2.1)	311 (2.1)	335 (2.2)	334 (2.2)		
Diabetes						< 0.001	0.130
No	59,371 (98.8)	14,988 (99.8)	14,946 (99.5)	14,882 (99.0)	14,555 (96.8)		
Yes	732 (1.2)	33 (0.2)	78 (0.5)	147 (1.0)	474 (3.2)		

Continuous variables were summarized using mean ± standard deviations or median (quartile 1, quartile 3), while categorical variables were expressed as n (%). BMI, body mass index; SBP, systolic blood pressure; DBP, diastolic blood pressure; FPG, fasting plasma glucose; TC, total cholesterol; HDL-C, high-density lipoprotein cholesterol; LDL-C, low-density lipoprotein cholesterol; ALT, alanine aminotransferase; AST, aspartate aminotransferase; BUN, blood urea nitrogen; Scr, creatinine; TG, triglyceride; TyG, triglyceride-glucose index; SMD,Standardized Mean Difference. An absolute SMD value < 0.1 typically indicates a negligible difference between groups.

The data presented in this table are derived from complete-case analysis. Cases with missing values were excluded from the present analysis and were not displayed.

### The association between TyG and diabetes risk

**Figure 2:**
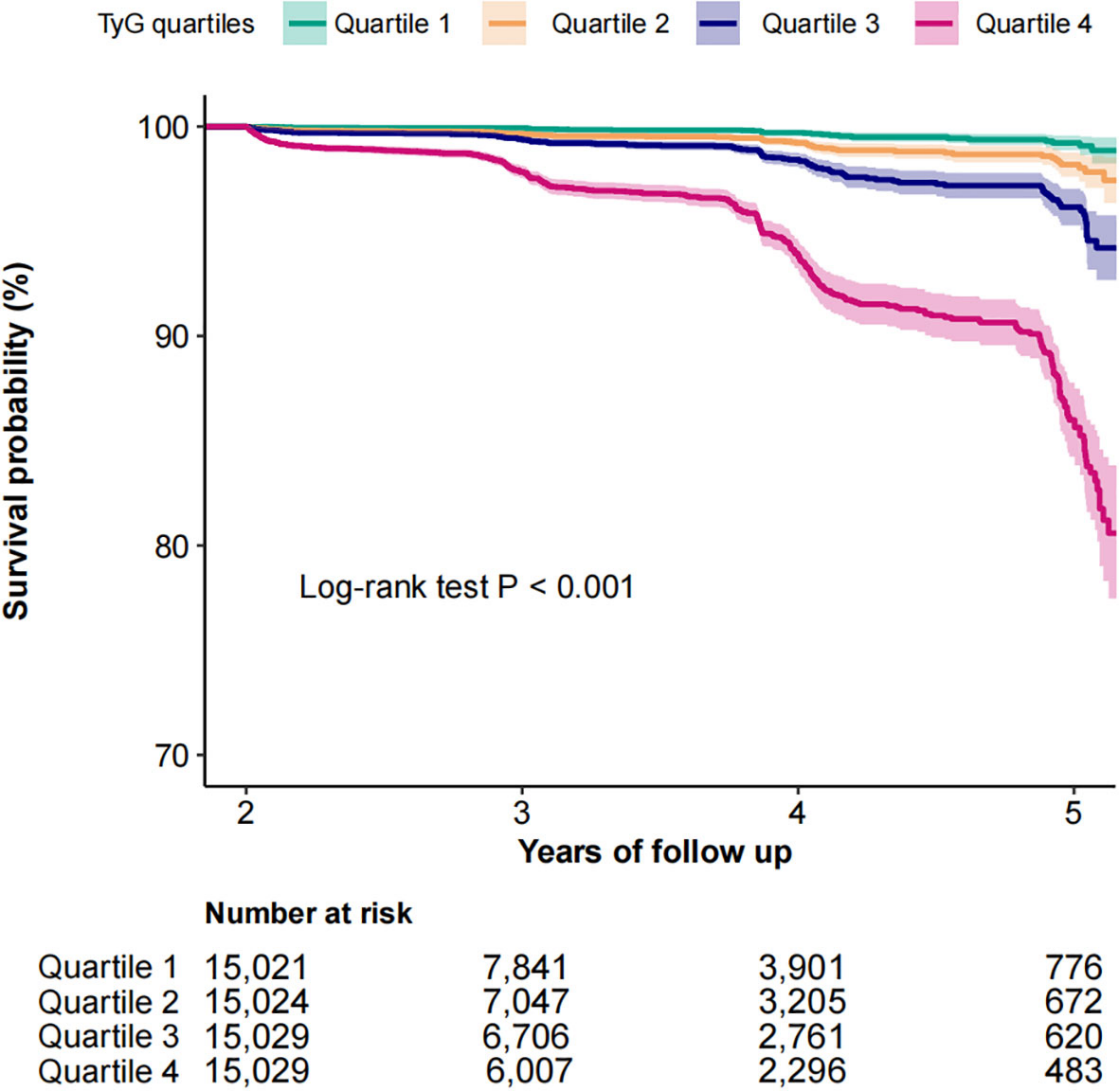
Kaplan-Meier curves for the probability of diabetes base on TyG quartiles among normolipidemic individuals.

The Kaplan-Meier survival analysis demonstrated a pronounced divergence in diabetes incidence across TyG quartiles, with the highest-risk cohort (quartile 4) exhibiting markedly reduced diabetes-free survival (Log-rank test *P* < 0.001, **Figure 2**). As shown in **Table 2**, multivariable Cox proportional hazards modeling revealed a strong positive association between TyG index elevation and diabetes risk. Each unit increment in TyG corresponded to a 10.10-fold escalation in diabetes hazard (HR: 10.10, 95% CI: 7.94–12.84; P < 0.001), maintaining statistical significance following comprehensive adjustment for clinical covariates (**Table 2**, Model 4). Notably, quartile-stratified analysis demonstrated a striking gradient effect. Participants in the TyG quartile 4 manifested a 19.76-fold heightened diabetes risk relative to the reference quartile 1 (HR: 19.76, 95% CI: 13.88–28.13; *P* < 0.001, **Table 2**, Model 1). This association retained both statistical significance and clinical relevance following multivariable adjustment, with quartile 4 subjects maintaining a 7.98-fold excess risk (HR: 7.98, 95% CI: 5.52–11.52; *P* < 0.001, **Table 2**, Model 4).

**Table 2:** The association between TyG and the risk of diabetes among individuals with normal lipid metabolism in different models.

TyG	Numbers	Event (%)	Model 1		Model 2		Model 3		Model 4	
			HR (95% CI)	*P* value	HR (95% CI)	*P* value	HR (95% CI)	*P* value	HR (95% CI)	*P* value
TyG	60,103	732 (1.2)	25.07 (19.94–31.53)	<0.001	12.37 (9.81–15.59)	<0.001	10.13 (7.97–12.88)	<0.001	10.10 (7.94–12.84)	<0.001
TyG quartiles										
Quartile 1	15,021	33 (0.2)	1.00 (Ref)		1.00 (Ref)		1.00 (Ref)		1.00 (Ref)	
Quartile 2	15,024	78 (0.5)	2.66 (1.77–4.00)	<0.001	2.07 (1.37–3.11)	<0.001	2.01 (1.33–3.02)	0.001	2.01 (1.34–3.03)	0.001
Quartile 3	15,029	147 (1.0)	5.40 (3.70–7.88)	<0.001	3.32 (2.27–4.86)	<0.001	3.08 (2.10–4.52)	<0.001	3.07 (2.09–4.51)	<0.001
Quartile 4	15,029	474 (3.2)	19.76 (13.88–28.13)	<0.001	9.47 (6.62–13.56)	<0.001	8.00 (5.54–11.56)	<0.001	7.98 (5.52–11.52)	<0.001
P for trend				<0.001		<0.001		<0.001		<0.001

DBP, diastolic blood pressure; SBP, systolic blood pressure; BMI, body mass index; FPG, fasting plasma glucose; TC, total cholesterol; HDL-C, high-density lipoprotein cholesterol; ALT, alanine aminotransferase; LDL-C, low-density lipoprotein cholesterol; AST, aspartate aminotransferase; BUN, blood urea nitrogen; Scr, creatinine; TG, triglyceride; TyG, triglyceride-glucose index; 95% CI, 95% confidence interval; HR, hazard ratio; Ref, reference.

Model 1: Not adjusted for any confounders.

Model 2: Adjusted for age, sex.

Model 3: Adjusted for age, sex, SBP, DBP, TC, BMI, LDL, HDL, ALT, AST, BUN, Scr.

Model 4: Adjusted for Model 3+family history of diabetes, smoking status, drinking status.

### Subgroup and sensitivity analyses

**Figure 3:**
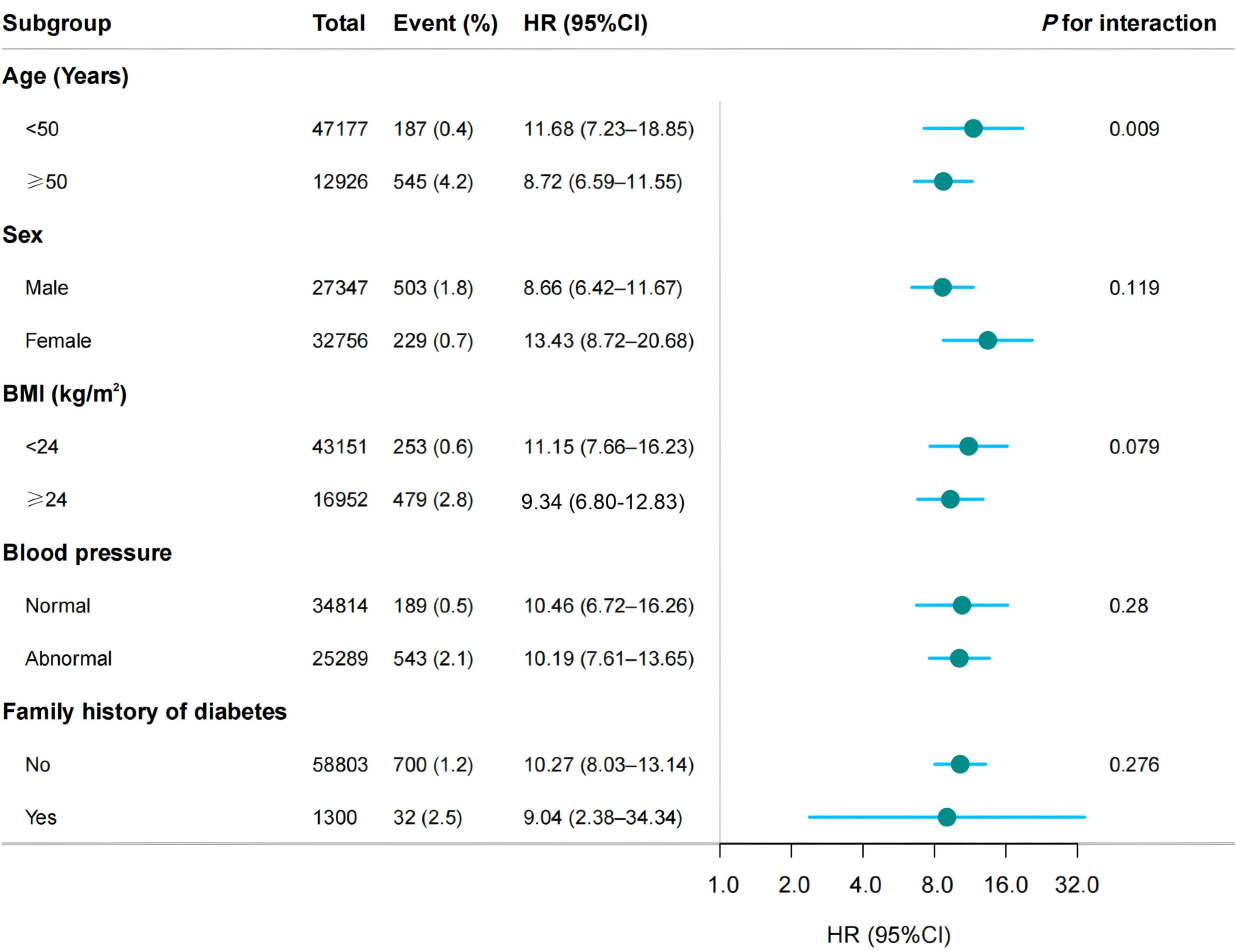
Forest plot of subgroup analysis of the association between TyG and the risk of diabetes among normolipidemic individuals. The model incorporated adjustments for age, sex, BMI, SBP, DBP, TC, LDL, HDL, ALT, AST, BUN, Scr, smoking status, drinking status, and family history of diabetes, except when family history of diabetes was the stratification variable.

As shown in **Figure 3**, subgroup analyses revealed consistently significant TyG-diabetes associations across all strata (*P* < 0.001), although the strength of association varied by subgroup. Most notably, age significantly modified this relationship (*P* for interaction = 0.01), with adults < 50 years old exhibiting markedly higher risk (HR: 11.68, 95%CI: 7.23–18.85) compared to their older counterparts (HR: 8.72, 95%CI: 6.59–11.55). While females showed numerically greater risk estimates than males (13.43 vs. 8.66), this difference did not reach statistical significance (*P* for interaction = 0.119). A similar non-significant trend was observed in non-overweight participants, who demonstrated marginally stronger associations compared to their overweight counterparts (HR: 11.15 vs. 9.34; *P* for interaction = 0.079). In contrast, hypertension status showed minimal differential effects (normotensive: 10.46 vs hypertensive: 10.19; *P* for interaction = 0.280), as did family history of diabetes (positive history: 9.04 vs negative: 10.27; *P* for interaction = 0.276). After excluding smokers or drinkers, the positive relationship between TyG and diabetes events remained stable. As showed in **Supplementary Table 2**, the results remained consistent in the analysis of the original data. In participants with baseline prediabetes, the TyG index remained significantly associated with the risk of diabetes. However, the effect strength was even greater in the normoglycemia group, highlighting its particular value in identifying high-risk individuals at a very early stage (HR: 2.99 vs. 4.81).

### Curve fitting and inflection point analysis

**Figure 4:**
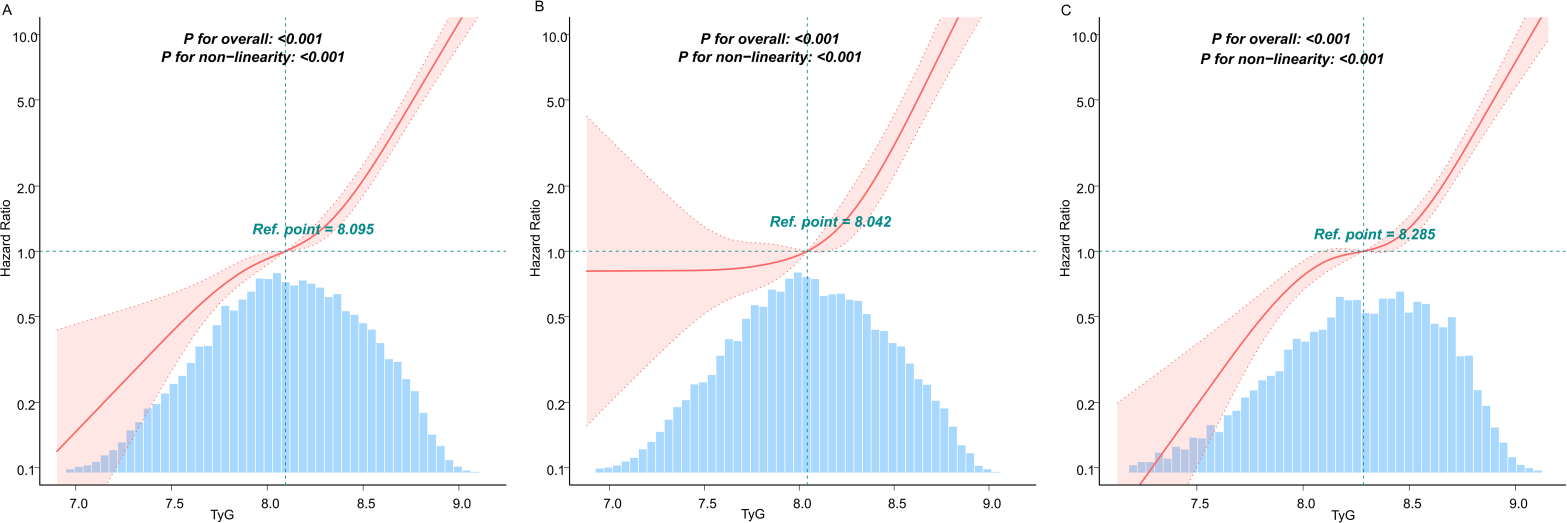
Analysis of the dose-response relationship between TyG and diabetes risk in people with normal lipid levels. **(A)** General population **(B)** Population <50 years old **(C)** Population ≥ 50 years old. The solid lines represent the multivariate-adjusted hazard ratios, while the dashed lines depict the 95% confidence intervals derived from restricted cubic spline regression. The horizontal dotted line indicates an hazard ratio of 1.0, serving as the reference point. The reference point for the TyG index was set at the median level within each respective population subgroup. The distribution of TyG population levels is depicted in the blue part of the bar chart. Cox regression analyses were adjusted for potential confounders, including age, sex, BMI, SBP, DBP, TC, LDL, HDL, ALT, AST, BUN, Scr, smoking status, and family history of diabetes. Extreme values of TyG (lowest 0.25%) were excluded from the analysis.

Given the statistically significant variations in the association between the TyG index and diabetes incidence across different age subgroups, we conducted further investigations using curve fitting analysis. Threshold effect analysis was employed to identify the TyG index’s turning points in various age groups. The curve fitting results revealed a pronounced J-shaped relationship between the TyG index and diabetes risk (*P* for nonlinearity <0.001; **Figure 4**). As shown in **Table 3**, a significant association between the TyG index and diabetes risk was observed when values were below 8.53. Strikingly, exceeding this threshold was associated with a dramatic 51.84-fold increase in diabetes risk (HR: 51.84, 95% CI: 24.83–108.24). In younger individuals (< 50 years old), no significant diabetes risk was detected when the TyG index remained below 8.20, but surpassing this level led to a sharp increase in risk (HR: 50.08, 95% CI: 20.91–119.96). In older adults (≥ 50 years old), even TyG levels below the inflection point (8.74) carried a measurable risk (HR: 4.89, 95% CI: 3.41–7.01), which escalated substantially when the threshold was exceeded (HR: 143.92, 95% CI: 29.04–713.27). The likelihood ratio test confirmed that the two-phase regression model provided significantly better fit (*P* < 0.001).

**Table 3:** Threshold effect of TyG on the incidence of diabetes among individuals with normal lipid metabolism.

The turning point for TyG	HR (95%CI)	*P* value
The general population		
< 8.53	3.61 (2.39–5.43)	< 0.001
≥ 8.53	51.84 (24.83–108.24)	< 0.001
Likelihood Ratio test		< 0.001
Age < 50 (years)		
< 8.20	1.33 (0.43–4.09)	0.6182
≥ 8.20	50.08 (20.91–119.96)	< 0.001
Likelihood Ratio test		< 0.001
Age ≥ 50 (years)		
< 8.74	4.89 (3.41–7.01)	< 0.001
≥ 8.74	143.92 (29.04–713.27)	< 0.001
Likelihood Ratio test		< 0.001

BMI, body mass index; SBP, systolic blood pressure; DBP, diastolic blood pressure; FPG, fasting plasma glucose; TC, total cholesterol; HDL-C, high-density lipoprotein cholesterol; LDL-C, low-density lipoprotein cholesterol; ALT, alanine aminotransferase; AST, aspartate aminotransferase; BUN, blood urea nitrogen; Scr, creatinine; TG, triglyceride; 95% CI, 95% confidence interval; HR, hazard ratio; TyG, triglyceride-glucose index.

The model adjusted for age, sex, SBP, DBP, BMI, TC, LDL, HDL, ALT, AST, BUN, Scr, smoking status and family history of diabetes. Extreme values of TyG (lowest 0.25%) were excluded from the analysis.

### Association between TyG and diabetes events in different age groups

RCS analysis (**Figure 4**) identified TyG = 8.53 as a statistically significant inflection point in the TyG-diabetes association (*P* for nonlinearity < 0.001). As showed in **Table 4**, when implementing this inflection point-derived TyG cutoff (≥ 8.53 vs < 8.53), the multiplicative interaction term reached statistical significance (*P* for interaction < 0.001). Age-stratified analyses revealed pronounced risk differentials: younger adults (< 50 years old) with TyG ≥ 8.53 had a 5.66-fold increased diabetes risk (HR: 6.66, 95% CI: 4.95–8.96) relative to the reference group, a 24.57-fold risk elevation in older adults (≥ 50 years old) with similarly elevated TyG levels compared to the group of younger adults (< 50 years old) with TyG < 8.53. The additive interaction measures demonstrated substantial effect modification, with a RERI of 11.98 (HR: 11.98, 95% CI: 7.60–16.36) and AP of 0.47 (HR: 0.47, 95% CI:0.38–0.56), suggesting that 47% of the excess diabetes risk resulted from the interaction between TyG and age.

**Table 4:** Multiplicative and additive interactions between different TyG and age groups on the risk of diabetes.

Variable	TyG status	HR (95% CI)	*P* for interaction	Additive interaction	
				RERI (95% CI)	AP (95% CI)
Age			<0.001	11.98 (7.60–16.36)	0.47 (0.38–0.56)
<50	Low	1.00 (Ref)			
	High	6.66 (4.95–8.96)			
≥50	Low	7.93(6.17–10.21)			
	High	25.57 (19.77–33.06)			

BMI, body mass index; SBP, systolic blood pressure; DBP, diastolic blood pressure; FPG, fasting plasma glucose; TC, total cholesterol; HDL-C, high-density lipoprotein cholesterol; LDL-C, low-density lipoprotein cholesterol; ALT, alanine aminotransferase; AST, aspartate aminotransferase; BUN, blood urea nitrogen; Scr, creatinine; TG, triglyceride; TyG, triglyceride-glucose index; 95% CI, 95% confidence interval; HR, hazard ratio; RERI, relative excess risk due to interaction; AP, attributable proportion.

The analysis adjusted for sex, BMI, SBP, DBP, TC, LDL, HDL, ALT, AST, BUN, Scr, family history of diabetes, smoking status, drinking status.

## Discussion

Our large-scale retrospective cohort study provides novel evidence regarding the TyG-diabetes association in normolipidemic populations. The analysis revealed a significant nonlinear positive correlation between TyG index and diabetes incidence, with particularly steep risk escalation observed at TyG levels exceeding 8.53 (HR: 51.84, 95%CI: 24.83–108.24). This association remained robust across all examined subgroups stratified by age, sex, BMI, blood pressure status, and family history of diabetes. Notably, we identified a significant positive interaction between advanced age and a high TyG index. The combination of both factors led to a dramatic rise in disease risk (HR = 25.57), an effect that was significantly greater than the additive effect of each individual factor. In assessing diabetes risk, it is essential to integrate both the TyG index and age, particularly regarding individuals aged 50 or above with elevated TyG levels, who should be classified into the highest-risk category and receive intensive intervention strategies.

Previous studies have extensively investigated the TyG-diabetes association but reported inconsistent findings. A systematic review and meta-analysis identified TyG index as a potential predictor for gestational diabetes mellitus in Asian women (33). Zhang et al. demonstrated a linear positive relationship between TyG index and GDM risk among Chinese singleton pregnancies (34). In American populations, studies revealed a nonlinear positive association, with significantly elevated diabetes risk when TyG exceeded 8.00 in men or 9.00 in women (21). Japanese research showed a U-shaped relationship between TyG and diabetes risk in normoglycemic individuals (35).The same conclusion was also found in the non-alcoholic fatty liver population (36). A dose-response meta-analysis of 14 cohort studies found progressively steeper risk escalation when TyG surpassed 8.6 (37), consistent with findings from a 15-year Chinese prospective study (inflection point = 8.51) (38). A study by Shan Yingqi et al. involving the general Chinese population aged 45 and above reported a TyG index inflection point at 8.516. The HR was 1.927 (95% CI: 1.31–2.83) below this threshold and 1.45 (95% CI: 1.21–1.74) above it (39).These findings align with those of Cao et al., who identified a similar inflection point (TyG = 8.73) in their study of the Chinese general population, with an HR of 1.95 (95% CI: 1.86–2.04) before the inflection point and 1.34 (95% CI: 1.27–1.42) thereafter (40).Among overweight/obese individuals (BMI ≥ 24 kg/m²), Sun Yongbing et al. observed a sharp increase in diabetes risk at TyG indices > 4.46, with older women (BMI 24–28 kg/m²) facing disproportionately higher risk at similar TyG levels (41). A study of elderly Chinese adults (aged ≥ 75 years old) also revealed a significant positive association between the TyG index and diabetes risk (42). Most existing studies have primarily examined the association between the TyG index and diabetes risk in general populations or high-risk groups, including individuals with obesity, hypertension, or advanced age. Emerging evidence suggests that even in populations traditionally considered at low risk for diabetes—such as non-obese young adults (aged < 50 years old)—the TyG index demonstrates a significant nonlinear positive association with diabetes incidence, with an inflection point at 7.3 (43). This finding is corroborated by similar research conducted among non-obese elderly populations (aged 40–69 years old) in South Korea, which likewise revealed a positive association (18). Previous studies have also shown that cumulative exposure to TyG index increases the risk of diabetes (44). In conclusion, the TyG index demonstrates significant potential as a predictive biomarker for diabetes risk. However, current evidence reveals considerable heterogeneity across studies regarding the TyG-T2DM association. Future research should focus on validating these findings through standardized methodologies and elucidating the precise mechanisms underlying this relationship.

Our study demonstrates a positive association between the TyG index and diabetes risk in individuals with normal lipid metabolism, as identified in a health examination cohort. The robustness of this association was consistently observed across various subgroups stratified by age, sex, BMI, blood pressure status, and family history of diabetes through comprehensive sensitivity analyses. These findings not only corroborate previous reports of the positive relationship between TyG and diabetes risk in normolipidemic populations, but more importantly, reveal a significant modifying effect of age on this association. Notably, the association between TyG index and diabetes risk demonstrated significant age-dependent heterogeneity, with a markedly stronger effect size in participants younger than 50 years old old (HR: 11.68, 95% CI: 7.23–18.85) compared to older individuals (HR: 8.72, 95% CI: 6.59–11.55). Notably, our threshold analysis identified age-specific optimal cutoff values for TyG index in diabetes risk stratification: 8.20 for individuals under 50 years old and 8.74 for those above 50 years old. In the younger population (< 50 years old old), the TyG risk threshold was lower (8.20), with diabetes risk increasing significantly only when TyG exceeded this level. In contrast, the older group (≥ 50 years old old) exhibited a higher TyG inflection point (8.74). Notably, this older cohort demonstrated measurable diabetes risk even at sub-threshold TyG levels. Interestingly, within the TyG range of 8.20–8.74, younger individuals experienced a more pronounced diabetes risk elevation per unit increase in TyG compared to their older counterparts. The observed age-dependent pattern demonstrates a critical divergence: although aging persists as an independent diabetes risk factor, the relative contribution of insulin resistance (quantified by TyG index) becomes more pronounced in younger individuals (< 50 years old old), indicating potential differences in disease pathophysiology between age cohorts. Traditionally, diabetes was considered a disease predominantly affecting elderly populations. However, accumulating epidemiological evidence indicates that the incidence of type 2 diabetes among young adults has increased several-fold in recent decades (45). The modern lifestyle characterized by excessive consumption of high-calorie diets, physical inactivity, and chronic psychosocial stress from work and social pressures may contribute to this concerning trend, even in individuals with apparently normal lipid profiles. In older adults (≥ 50 years old old), diabetes risk escalates progressively with rising TyG levels, showing measurable risk even below the threshold. However, when TyG exceeds 8.74, the hazard ratio surges dramatically to 143.92 - nearly threefold higher than in younger populations (HR: 50.08). These findings carry important clinical implications. For younger adults, TyG monitoring should focus on the 8.20 threshold for preventive intervention. Older populations require vigilant metabolic surveillance even at sub-threshold TyG levels (8.74), with aggressive management recommended when exceeding this cutoff. The striking magnitude of risk differential (143.92 vs 50.08) underscores the necessity of age-tailored approaches when implementing TyG-based diabetes risk stratification in clinical practice. Further prospective cohort studies with serial measurements are warranted to validate these cutoff values and to elucidate the dynamic relationship between TyG index and diabetes risk progression. Mechanistic studies are also needed to clarify the pathophysiological basis underlying the observed age-dependent differences in the TyG-diabetes association, particularly in younger populations with normal lipid metabolism who are increasingly recognized as an important target group for diabetes prevention.

Although our retrospective cohort study design cannot establish causal relationships between the TyG index and diabetes incidence, several well-established pathophysiological mechanisms may help interpret our findings. Primarily, insulin resistance represents one of the fundamental pathological mechanisms underlying type 2 diabetes development (46). The TyG index serves as a reliable surrogate marker of insulin resistance, reflecting its severity. Chronic insulin resistance induces progressive β-cell dysfunction, ultimately leading to impaired insulin secretion, elevated blood glucose levels, and consequently increased diabetes risk. From a pathophysiological perspective, insulin resistance exerts multifaceted metabolic effects (1): it enhances lipolysis in adipose tissue, resulting in elevated circulating lipid levels (2); it impairs lipoprotein metabolism, delaying triglyceride clearance; and (3) it reduces peripheral glucose uptake and utilization efficiency, collectively contributing to systemic metabolic dysregulation. Notably, the interplay between insulin resistance and metabolic disturbances may exacerbate diabetes risk through activation of inflammatory pathways. This mechanistic link is supported by emerging evidence demonstrating significant association between the TyG index and various inflammatory biomarkers. Notably, a key outcome of compensatory insulin resistance is hyperinsulinemia. Accumulating evidence indicates that hyperinsulinemia is associated with a range of long-term pathological changes, independent of blood glucose levels. These include promoting atherosclerosis and increasing cardiovascular risk, accelerating cellular senescence, enhancing susceptibility to certain cancers, and contributing to neurodegenerative disorders such as Alzheimer’s disease (47–50). Early identification of insulin resistance is vital not only to prevent the onset of type 2 diabetes but also to address the broader spectrum of metabolic and cardiovascular diseases associated with this condition. By recognizing and managing insulin resistance early, healthcare providers can implement targeted interventions to reduce the risk of progression to more severe health issues, thereby improving patient outcomes and reducing the burden of chronic diseases.

Several limitations of this study warrant careful consideration. First, as a retrospective observational cohort study, the identified associations do not imply causality and may be influenced by unmeasured confounding variables. Although we rigorously adjusted for all available potential confounders in our multivariate models, residual confounding remains possible. To assess the robustness of our findings, comprehensive sensitivity analyses were performed; these evaluations consistently confirmed the stability of the primary outcomes. Second, the absence of key glycemic parameters, including postprandial glucose levels and hemoglobin A1c measurements, represents an important limitation. Our diabetes definition relied solely on fasting glucose levels and self-reported diagnoses, which may have introduced ascertainment bias and potentially led to underestimation of both diabetes incidence and effect sizes. Third, the mean follow-up duration of 3.06 years may have limited our study’s statistical power to detect diabetes events. This relatively short observation period could have constrained our ability to fully characterize the TyG-diabetes association. Fourth, the data for our study were derived from a previous investigation, which provided only the processed dataset. Consequently, we were unable to assess the selection bias that may have been introduced during the exclusion of participants from the original cohort (n = 685,277) to arrive at the shared dataset (n = 211,833). Despite this limitation, our study retains significant value, as it offers an in-depth longitudinal analysis of a well-defined, large-scale healthy population—delivering unique insights into the research question. Although restricted by the lack of access to the primary data, we employed internal comparisons to evaluate potential biases within the available data to the greatest extent possible. Sensitivity analyses further demonstrated the robustness of our findings. Additionally, while our study benefited from a large sample size, the generalizability of our findings may be limited to Chinese populations with normal lipid metabolism. Caution should be exercised when extrapolating these results to other demographic groups.

## Conclusions

Our 60,103 longitudinal analysis demonstrates that even in individuals with normal lipid metabolism, elevated levels of the TyG index significantly increase the risk of diabetes. This association exhibits a nonlinear dose-response relationship and is significantly age-dependent, with young adults (< 50 years old old) showing a markedly higher sensitivity to the diabetes risk associated with the TyG index compared to older adults. These findings challenge traditional screening approaches, indicating that a normal lipid profile does not rule out the risk of diabetes, particularly in younger individuals. Assessing the TyG index can enhance the detection rate of early-stage diabetes in those with normal metabolic profiles and underscores the importance of age-specific risk stratification. Moreover, the stronger association observed in young adults suggests different pathophysiological mechanisms or varying exposure to modern lifestyle risk factors, necessitating targeted preventive measures for this population.

## Data Availability

The datasets presented in this study can be found in online repositories. The names of the repository/repositories and accession number(s) can be found in the article/**Supplementary Material**.
