# Supplementary material for: Threshold effect and age interaction of TyG index on diabetes incidence in normolipidemic population: a multicenter cohort study
Source: Front Endocrinol (Lausanne). 2025 Nov 3;16:1645344. doi: 10.3389/fendo.2025.1645344 (PMC12620258; doi:10.3389/fendo.2025.1645344)
Supplement: Supplementary file 1 [file Table1.docx]

**Supplementary Table 1 Missing data distribution for key variables**

| Variables | Numbers | Percentage (%) |
| --- | --- | --- |
| ALT | 242 | 0.40 |
| AST | 34,169 | 56.85 |
| BUN | 1,343 | 2.23 |
| Scr | 814 | 1.35 |
| SBP | 6 | 0.01 |
| DBP | 6 | 0.01 |
| Smoking status | 44,157 | 73.47 |
| Drinking status | 44,157 | 73.47 |

Note: SBP, systolic blood pressure; DBP, diastolic blood pressure; ALT, alanine aminotransferase; AST, aspartate aminotransferase; BUN, blood urea nitrogen; Scr, creatinine.
