# Supplementary material for: Threshold effect and age interaction of TyG index on diabetes incidence in normolipidemic population: a multicenter cohort study
Source: Front Endocrinol (Lausanne). 2025 Nov 3;16:1645344. doi: 10.3389/fendo.2025.1645344 (PMC12620258; doi:10.3389/fendo.2025.1645344)
Supplement: Supplementary file 2 [file Table2.docx]

**Supplementary Table 2 The association between TyG and diabetes envents in different sensitivity analyses**

| **Population** | **Sensitivity 1** | | **Sensitivity 2** | | **Sensitivity 3** | | **Sensitivity 4** | | **Sensitivity 5** | |
| --- | --- | --- | --- | --- | --- | --- | --- | --- | --- | --- |
|  | **HR (95% CI)** | ***P* value** | **HR (95% CI)** | ***P* value** | **HR (95% CI)** | ***P* value** | **HR (95% CI)** | ***P* value** | **HR (95% CI)** | ***P* value** |
| TyG | 8.53 (4.47–16.26) | < 0.001 | 9.53 (5.31–17.07) | < 0.001 | 4.81 (3.21–7.21) | <0.001 | 2.99 (2.24–4.00) | <0.001 | 10.35 (8.10–13.23) | <0.001 |
| TyG quartiles |  |  |  |  |  |  |  |  |  |  |
| Quartile 1 | 1.00 (Ref) |  | 1.00 (Ref) |  | 1.00 (Ref) |  | 1.00 (Ref) |  | 1.00 (Ref) |  |
| Quartile 2 | 6.56 (1.46–29.55) | 0.016 | 5.00 (1.45–17.17) | 0.012 | 1.13 (0.64–1.97) | 0.678 | 1.29 (0.93–1.79) | 0.121 | 1.85 (1.22–2.80) | 0.004 |
| Quartile 3 | 10.73 (2.51–45.95) | 0.002 | 8.55 (2.58–28.38) | 0.001 | 1.88 (1.13–3.13) | 0.015 | 1.77 (1.30–2.41) | <0.001 | 2.96 (2.01–4.35) | <0.001 |
| Quartile 4 | 21.28 (5.08–89.10) | < 0.001 | 17.16 (5.27–55.92) | < 0.001 | 3.63 (2.22–5.94) | <0.001 | 2.68 (1.98–3.62) | <0.001 | 7.76 (5.37–11.23) | <0.001 |
| P for trend |  | < 0.001 |  | < 0.001 |  | < 0.001 |  | < 0.001 |  | < 0.001 |

Note: BMI, body mass index; SBP, systolic blood pressure; DBP, diastolic blood pressure; FPG, fasting plasma glucose; TC, total cholesterol; HDL-C, high-density lipoprotein cholesterol; LDL-C, low-density lipoprotein cholesterol; ALT, alanine aminotransferase; AST, aspartate aminotransferase; BUN, blood urea nitrogen; Scr, creatinine; TG, triglyceride; TyG, triglyceride-glucose index; 95% CI, 95% confidence interval; HR, hazard ratio

Sensitivity 1: Results for nonsmokers (n=13,016). Sensitivity 2: Results for nondrinkers (n=13,190). Sensitivity 3: Results for individuals with baseline

normoglycemia (n=54,583). Sensitivity 4: Results for individuals with baseline prediabetes (n=5,520). Sensitivity 5: Results based on the original data (n=58,554). The model adjusted for age, sex, BMI, SBP, DBP, TC, LDL, HDL, ALT, AST, BUN, Scr, smoking status (not for the nonsmokers), drinking status (not for the nondrinkers) and family history of diabetes.
